# Minicells as a Damage Disposal Mechanism in Escherichia coli

**DOI:** 10.1128/mSphere.00428-18

**Published:** 2018-09-19

**Authors:** Camilla U. Rang, Audrey Proenca, Christen Buetz, Chao Shi, Lin Chao

**Affiliations:** a University of California San Diego, La Jolla, California, USA; University of Wyoming

**Keywords:** *Escherichia coli*, aging, antibiotic resistance, minicells, oxidative damage

## Abstract

Many bacteria produce small, spherical minicells that lack chromosomal DNA and therefore are unable to proliferate. Although minicells have been used extensively by researchers as a molecular tool, nothing is known about why bacteria produce them. Here, we show that minicells help Escherichia coli cells to rid themselves of damaged proteins induced by antibiotic stress. By comparing the survival and growth rates of wild-type strains with the E. coli *ΔminC* mutant, which produces excess minicells, we found that the mutant was more resistant to streptomycin. To determine the effects of producing minicells at the single-cell level, we also tracked the growth of *ΔminC* lineages by microscopy. We were able to show that the mutant increased the production of minicells in response to a higher level of the antibiotic. When we compared two sister cells, in which one produced minicells and the other did not, the daughters of the former had a shorter doubling time at this higher antibiotic level. Additionally, we found that minicells were more likely produced at the mother’s old pole, which is known to accumulate more aggregates. More importantly, by using a fluorescent IbpA chaperone to tag damage aggregates, we found that polar aggregates were contained by and ejected with the minicells produced by the mother bacterium. These results demonstrate for the first time the benefit to bacteria for producing minicells.

**IMPORTANCE** Bacteria have the ability to produce minicells, or small spherical versions of themselves that lack chromosomal DNA and are unable to replicate. A minicell can constitute as much as 20% of the cell’s volume. Although molecular biology and biotechnology have used minicells as laboratory tools for several decades, it is still puzzling that bacteria should produce such costly but potentially nonfunctional structures. Here, we show that bacteria gain a benefit by producing minicells and using them as a mechanism to eliminate damaged or oxidated proteins. The elimination allows the bacteria to tolerate higher levels of stress, such as increasing levels of streptomycin. If this mechanism extends from streptomycin to other antibiotics, minicell production could be an overlooked pathway that bacteria are using to resist antimicrobials.

## Abstract (precis)

Bacteria have the ability to produce minicells, or small spherical versions of themselves that lack chromosomal DNA and are unable to replicate. A minicell can constitute as much as 20% of the cell’s volume. Although molecular biology and biotechnology have used minicells as laboratory tools for several decades, it is still puzzling that bacteria should produce such costly but potentially nonfunctional structures. Here, we show that bacteria gain a benefit by producing minicells and using them as a mechanism to eliminate damaged or oxidated proteins. The elimination allows the bacteria to tolerate higher levels of stress, such as increasing levels of streptomycin. If this mechanism extends from streptomycin to other antibiotics, minicell production could be an overlooked pathway that bacteria are using to resist antimicrobials.

## INTRODUCTION

Minicells are small spherical cells that bud off the poles of bacteria. They were first identified in bacilli (1) and have subsequently been reported in both Gram-negative and Gram-positive bacteria and chloroplasts (2–7). Because bacterial poles are chromosome free, minicells have no chromosomal DNA (8). However, they harbor membranes, ribosomes, RNA, protein, and plasmid DNA. As a result, minicells cannot replicate but are capable of other cellular functions (9). Before the advent of molecular tools such as PCR and green fluorescent protein (GFP) reporters, minicells were widely used as a vehicle for studying a variety of cellular processes, including protein synthesis and viral infections, and for isolating high-purity plasmid DNA from bacterial cells (10–20). More recently, there has been a renewed interest in using minicells as a smaller platform for visualizing macromolecule function and as a nonproliferating vector to deliver DNA, cancer drugs, and vaccines to a variety of host cells (21–32). However, despite all these benefits that minicells have provided to biological research, little is known about whether minicells provide any advantage to the bacteria that produce them. Why produce a small cell devoid of DNA and incapable of future replication?

Here, we propose and test the hypothesis that minicells provide an advantage to bacteria by serving as a mechanism for ridding the cell of damage. Nongenetic damage caused by oxidation and other stresses to macromolecules, organelles, tissues, and organisms is considered to be a major cause of biological aging (33). The central role played by oxidative damage suggests that the evolution of aerobic respiration exacted a high price. Many mechanisms may have evolved to cope with such high rates of damage. Unlike DNA damage, which induces heritable mutations, nongenetic damage can be diluted by turnover. Macromolecules that are more vulnerable to damage could be replaced more frequently. Damaged proteins are commonly grouped with the help of chaperones and organized into protein aggregates. Proteins in the aggregates can then be repaired or disassembled (34). The history of the discovery of minicells suggests that bacteria may have evolved an additional mechanism. The first reports of minicells in Escherichia coli came from studies that had subjected the cells to stress, such as high temperatures and growth inhibitors such as urethane (2, 5, 35). The *minC* mutation, which elevates the rate of minicell production, was isolated in E. coli that was being screened only to tolerate high doses of ionizing radiation (8). Because heat, growth inhibitors, and radiation can all cause damage to cells, the correlation between stress and the first observations of minicells clearly points to a possible causal relationship. Another possible association between minicells and damage is indicated by the molecular mechanism used by bacterial cells to control cell division and the formation of protein aggregates.

Cell division in rod-shaped bacteria such as E. coli is affected greatly by the *minCDE* operon and the FtsZ protein (36–39). The septum that divides a bacterial cell is determined by the position of the Z-ring formed by polymerization of FtsZ subunits (40, 41). The bacterial cell divides in the middle because the Z-ring is generally positioned at the center of the cell. If the Z-ring is positioned at the pole, a minicell is produced (39, 42). The central location of the Z-ring results from its interaction with the MinCDE complex (9). The MinD subunits initiate the complex by binding to a pole and polymerizing. The MinC subunits follow by binding to MinD polymers. Because MinC inhibits the polymerization of FtsZ, the Z-ring is excluded from the pole (43). The MinCD clusters are prevented from spreading into the center of the cell by MinE, which displaces MinC from MinD and also triggers the release of MinD from the membrane (44–46). The released MinC and MinD are then free to disperse to the other pole and initiate a new round of polymerization. In E. coli, MinC travels back and forth between the poles and clears the cell center for the formation of the Z-ring (38, 47–50). The Δ*minC* mutant makes minicells because the MinCDE complex fails to be completed at the poles. Minicells could also be produced in a cell with nonmutated *minC* if a regulatory failure cleared the MinCDE complex from the poles. If stress increases the chances of regulatory failure, minicell production could become associated with a buildup of cell damage. Most importantly, because damaged proteins assembled into aggregates tend to be associated with the polar end of bacteria (51–54), they could be ejected by being contained in minicells.

A historical difficulty with investigating if minicells are beneficial to bacteria results from the fact that wild-type cells produce them very infrequently under standard laboratory conditions (2). At those low rates, any possible benefit of producing minicells would be difficult to quantify. Attempts, including by our laboratory, to identify environmental agents that could trigger a higher rate have not been successful. We chose, therefore, to examine whether the higher minicell production by the *ΔminC* mutant of E. coli provided any advantage relative to a coisogenic wild type. The use of a mutation that enhances a desired phenotype to study its potential benefit can be instructive. For example, the first studies that examined whether mutations could be more beneficial or deleterious in bacteria relied on mutator strains with elevated mutation rates (55–57). The mutator loci provided an evolutionary advantage, and follow-up studies found that bacterial lineages in both wild and laboratory populations can evolve between mutator and nonmutator states (58, 59). Thus, the elevated rate turned out to be not just a convenient tool for magnifying the effect of the phenotype but rather a real adaptive evolutionary state. Whether minicell-producing mutants could evolve as an adaptive state is beyond the scope of this study, but we have taken the first step by examining whether and how the elevated production of minicells provides an advantage to bacteria. Our results show that the production of minicells under stressful conditions, rather than just being a pathological aberration of the cell cycle, benefits E. coli by helping the cells rid themselves of damage.

## RESULTS

The description of the materials and methods used in our study is abbreviated in this section in order to not overload the presentation of the results. A more detailed description is presented in Materials and Methods. We note that statistical significance in the figures is presented according to the standard notation of *, **, and *** to denote *P* values less than 0.05, 0.01, and 0.001, respectively. The *P* values and sample sizes are always presented in the figure legends. Except for the box plots in Fig. 8A and B, error bars are always presented as standard errors of the means. If error bars are not visible, it is because they are smaller than the graphing points and therefore not visible.

### Control and induced damage rates.

To examine the relationship between damage and minicells in E. coli, we subjected growing wild-type and Δ*minC* E. coli to a control level of 0 and a treatment level of 6 µg streptomycin ml^−1^. The wild type carried a Δ*malT* deletion, which was primarily used as a genetic marker to identify the strain. Both the Δ*malT* wild-type and Δ*minC* strains were from the Keio collection, which is a set of E. coli K-12 strains with unique single-gene deletions introduced with the λ Red recombinase protocol (60). Because all Keio strains were created by the same protocol from the same original strain, they are otherwise isogenic. Thus, the Keio Δ*malT* strain served as a wild-type and complementary control for the Δ*minC* knockout. The most common secondary mutations caused by the λ Red system are partial duplications, but 98.3% of the Keio strains, including the strains used in this study, have been found not to harbor any (61) (see Materials and Methods). The possible presence of secondary mutations is also addressed by a later experiment (see “Effect of minicell production on doubling times” below). A concentration of 6 µg streptomycin ml^−1^ was chosen because it is sublethal and offers an opportunity for the bacteria to survive the antibiotic challenge. At 6 µg ml^−1^, streptomycin increases the generation of damaged proteins by inducing mistranslations (62) and also making the cells more sensitive to oxidative damage (63). The damaged proteins are often assembled into aggregates or inclusion bodies, which can be visualized by microscopy and GFP fluorescence markers. The M9 minimal medium was used for all microscopy experiments because the autofluorescence of broth medium interfered with the detection of GFP.

**FIG 1:**
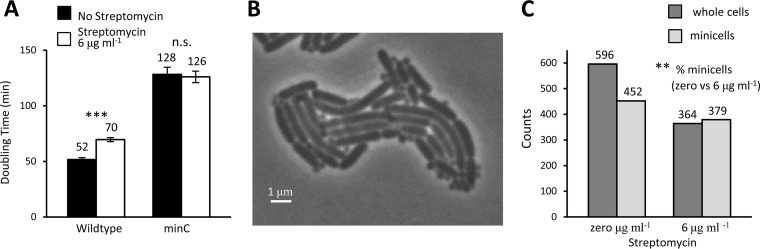
Doubling times and minicell production by Δ*minC* E. coli with streptomycin. Doubling times were obtained by tracking single cells from birth to division into two proliferating cells from the phase-contrast images. The production of minicells was not regarded as division since minicells lack chromosomal DNA and cannot grow and divide. (A) Average doubling times of Δ*minC* strain and wild-type Δ*malT* control measured by microscopy on agar pads at 0 and 6 µg streptomycin ml^−1^. The doubling times of the wild type increased significantly in going from the lower to the higher concentration (unpaired *t* test; *n* = 144 and 163; *P*  < 0.001). The doubling times of the Δ*minC* bacteria were not significantly different at the two streptomycin levels (unpaired *t* test; *n* = 186 and 185; *P*  = 0.79). (B) Phase-contrast microscopy image of a growing colony of Δ*minC* E. coli on an agar pad with 6 µg streptomycin ml^−1^ to illustrate the presence of minicells. (C) Numbers of whole cells and minicells in Δ*minC* colonies on agar pads with 0 and 6 µg streptomycin ml^−1^. The frequency (or percentage) of minicells at 6 µg streptomycin ml^−1^ was significantly greater than at 0 µg by a randomization test for differences (*P* = 0.0013; StatKey statistical package). Error bars show standard errors of the means. *, **, and *** denote *P* values less than 0.05, 0.01, and 0.001, respectively. n.s., not significant.

By using phase-contrast time-lapse microscopy to track bacteria in colonies, we measured and compared the doubling times of individual wild-type and *ΔminC* cells at 0 and 6 µg streptomycin ml^−1^. Because the cells used to start the colonies were grown in the absence of streptomycin, the 6-µg streptomycin ml^−1^ treatment challenges them with an antibiotic stress. Thus, the wild-type cells not only grew more slowly at 6 µg ml^−1^ than at 0 µg (Fig. 1A), they also stopped growing after 3 to 4 divisions. All doubling time measurements were based on cells that completed division. However, although the wild-type cells were affected by the antibiotic, the Δ*minC* E. coli cells were not. The Δ*minC* cells grew more slowly in general than the wild-type cells, possibly because of the time needed to make minicells, but they were able to sustain their growth at 6 µg streptomycin ml^−1^ with no limitation to the number of cell divisions (Fig. 1A). More importantly, the doubling times of Δ*minC* cells at 0 and 6 µg streptomycin ml^−1^ were not significantly different (*P* = 0.79) (Fig. 1A). Doubling times for Δ*minC* cells were scored from birth to division, and the production of minicells was not counted as a division. The addition of streptomycin affected the rate of minicell production by Δ*minC* E. coli. By comparing the numbers of minicells produced per whole cell, as illustrated in Fig. 1B, streptomycin was found to increase significantly the production rate (Fig. 1C). While at 0 µg streptomycin ml^−1^ the ratio of minicells to whole cells was 0.76, the ratio increased to above 1.0 at 6 µg ml^−1^. The mechanism for this increase is unknown, but the response could well be linked to cellular stress. No minicells were observed in our colonies of wild-type E. coli. The wild-type cells are reported to produce minicells at a rate of about one “among several thousand cells” or slightly less than 0.001 (2), which is too small to be detected with our colony sizes.

### Production of minicells by different poles and daughters.

**FIG 2:**
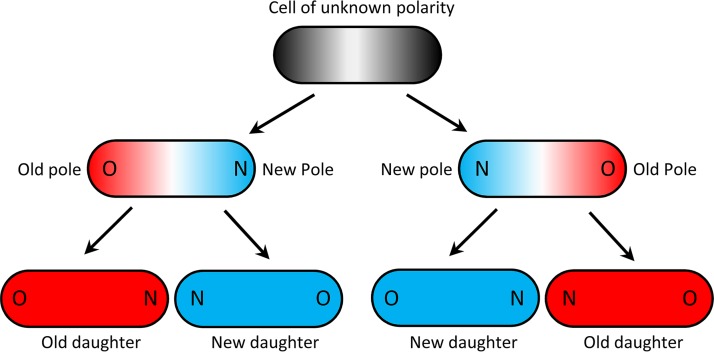
Assignment of old and new poles and daughters in E. coli. The blue and red colors denote new and old, respectively, for both poles and daughters. Because the division plane in a rod-shaped bacterium such as E. coli cuts the cell at the midpoint of the long axis, the poles formed at the plane are new and the distal poles are old. If the polarity of the first cell is unknown, old and new poles can be determined after one division, but the assignment of old and new daughters requires two divisions. The polarity of the first cell can be determined if its genealogy can be tracked backward one or more generations. Note that the bottom four daughters are colored red and blue to designate them as old and new daughters, but their old and new poles are identified by O and N.

To determine if minicells and damage could be associated, we explored the production of minicells by the old poles and old daughters of Δ*minC* bacteria. The ends of rod-shaped bacteria such as E. coli are polarized as new and old because cell division occurs at the septum located at the middle of the long axis. The ends or poles at the septum are newly synthesized and designated new. Thus, all E. coli cells have a new and an old pole. The two daughters produced by the division of a mother E. coli cell are additionally designated old and new, depending on which one acquires the mother’s old and new pole (Fig. 2). Note that while the new and old poles of the daughters can be determined by time-lapse microscopy immediately after the division of the mother bacterium, the identification of the old and new daughters requires the tracking of the cells for one more division. More importantly, damaged proteins are often associated with the old pole (51–54) and old daughters (64–67) of the bacteria.

**FIG 3:**
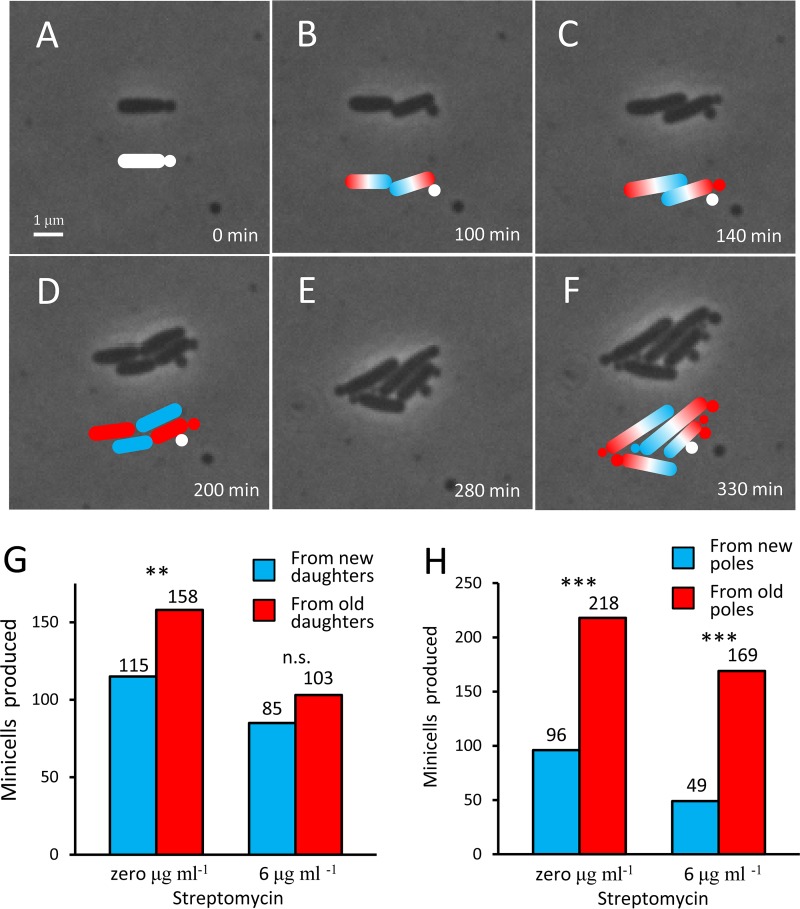
Minicell production by poles and daughters. (A to F) Phase-contrast microscopy images of a growing colony of Δ*minC* E. coli on an agar pad with 6 µg streptomycin ml^−1^ to illustrate the production of minicells by poles and daughters. Colored cartoons are drawn to identify new (blue) and old (red). Because the cell and the minicell that it produces in panel A are of unknown polarity, they are cartooned white. In panel B, the cell division establishes the old and new poles. Thus, the minicell produced in panel C is cartooned red to indicate that it was produced from an old pole. In panel F, a total of seven minicells are identified. Five (red) came from old poles, and one (blue) came from a new pole. Note that minicells could have been assigned instead to whether they came from an old or new daughter. Panel D illustrates the time when old and new daughters can be determined. (G) Production of minicells from old versus new daughters at 0 and 6 µg streptomycin ml^−1^. The percentage of minicells that came from old daughters was 58% and 55% at the two concentrations, but only the first measurement was significantly different from a null model of 50% probability (chi-square; *P* = 0.0093 and 0.189). However, the trend was sufficiently strong such that significance was increased by pooling the samples from 0 and 6 µg ml^−1^ (chi-square; *P* = 0.0045). (H) Production of minicells from old versus new poles at 0 and 6 µg streptomycin ml^−1^. Minicells came from old poles at the two concentrations, respectively, with a probability of 69% and 78%. Both probabilities were significantly greater than a null of 50% (chi-square; *P* < 0.001 and < 0.001). *, **, and *** denote *P* values less than 0.05, 0.01, and 0.001, respectively.

We first tracked by microscopy the production of minicells from old and new daughters grown at 0 and 6 µg streptomycin ml^−1^. By tracking growing cells under the microscope, it is possible to determine old and new daughters and old and new poles, as illustrated in Fig. 3A to F. The exemplified bacterium begins as a minicell-producing single cell with unknown polarity at *t* = 0 min (Fig. 3A); divides twice and grows to a colony of 4 cells, consisting of two old and two new daughters (Fig. 3D); and has produced 5 minicells from old poles and one from new poles by 330 min (Fig. 3F). By monitoring numerous colonies at both concentrations of streptomycin, we noted the trend that minicells were produced more frequently by an old daughter, but the difference was significant only at 0 µg streptomycin ml^−1^ (Fig. 3G). If the Fig. 3G data were pooled, they remained significant (*P* = 0.0045; *n* = 461). A stronger trend was observed for the production of minicells by the old and new poles of a cell. At both 0 and 6 µg streptomycin ml^−1^, whenever a minicell was produced, it came more often from the old pole (Fig. 3H). When the same data are expressed as probabilities, the chance that a minicell came from the old pole was 69 and 78% at the two streptomycin concentrations, respectively. Thus, the origination of minicells appears to be linked with old poles and old daughters, both of which have been shown to accumulate damage at a higher rate (51). The link was stronger for old and new poles, rather than daughters, because new daughters do sometimes make minicells, but they will then more likely make them from their old pole.

### Minicells and the ejection of damage.

To examine whether the link between minicell production and damage had functional consequences, we tagged the cell wall and aggregates of damaged proteins with fluorescent markers and used microscopy to track their fate near the old poles of cells. Both the cell wall and protein aggregates were monitored because they could harbor different types of damage. Because the poles of E. coli are inert (68, 69), the turnover of polar cell wall is slow and damage could accumulate. Because minicells are produced at the poles, they could harbor the older cell walls, but the origin of the cell wall of minicells had never been verified. Determining whether any cell walls and protein aggregates were preferentially ejected with minicells would allow us to evaluate more completely the potential benefit of producing minicells.

**FIG 4:**
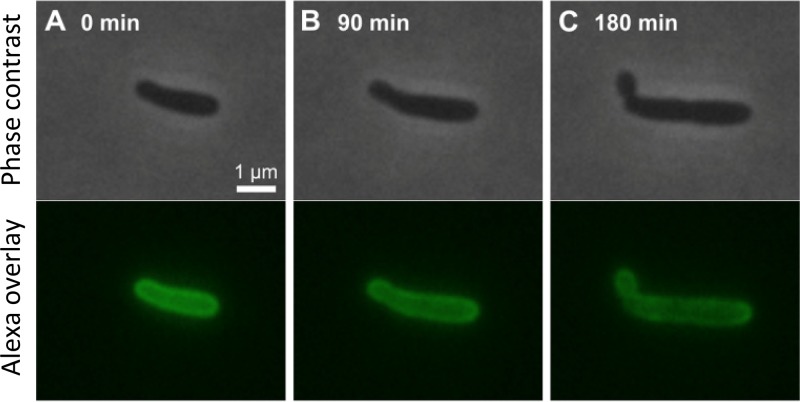
Time-lapse microscopy of a growing single cell with fluorescently labeled cell wall. (A to C) Phase-contrast and fluorescent images (upper and lower rows, respectively) of a Δ*minC* E. coli cell on an agar pad at 90-min intervals. The wall of the cell was initially pulse-labeled with Alexa Fluor 488. Because, after the pulse-label, new cell wall synthesized during growth is not fluorescent, the label serves as a marker for the original cell wall. From the first to the last image, the cell elongates from approximately 2.5 to 4 μm over 180 min, an 80% increase in length. Note that while the middle cylinder of the Alexa image at 180 min (C) is darker, the pole and minicell are brighter. Thus, new growth appears to be concentrated in the middle cylinder of the cell and the minicell receives the older cell wall.

To monitor the cell wall dynamics during and after minicell release, growing *ΔminC* E. coli was pulse-labeled with Alexa Fluor 488. After the cells walls were rendered fluorescent by the incorporation of Alexa during cell growth, the excess Alexa was washed off, and cell growth was monitored in colonies by time-lapse fluorescence microscopy. With the excess removed, newly synthesized cell walls lack fluorescence and will appear darker. From our time-lapse videos (Fig. 4), it was evident that new cell wall components were being added primarily to the middle cylinder of the cell. Whereas the 0-min Alexa frame showed a much more uniform fluorescence over the entire cell (Fig. 4A), the 180-min Alexa frame revealed that the midsection of the cell was becoming less bright (Fig. 4C). At 180 min, it was the pole and the minicell that were brighter. Thus, the minicell did not acquire newly synthesized cell walls but was rather allocated existing cell wall from the mother cell.

**FIG 5:**
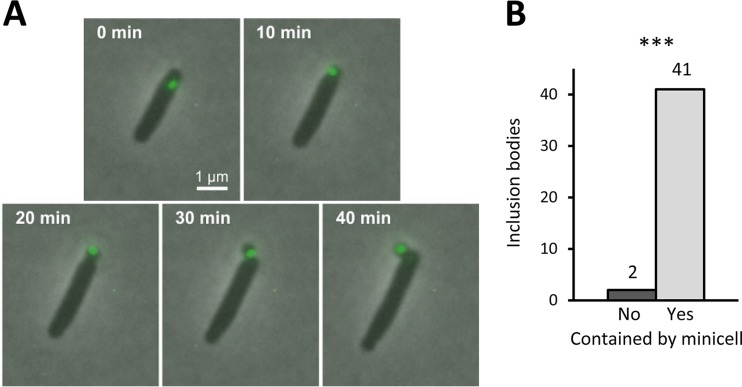
Capture and ejection of inclusion bodies by minicells. (A) Time-lapse microscopy of an inclusion body in a growing E. coli cell about to produce a minicell. The inclusion body was tagged with an *IbpA-yfp* fusion (51) and tracked by fluorescence microscopy. IbpA is a small chaperone that facilitates the refolding of damaged proteins and is therefore associated with inclusion bodies in E. coli (70). As shown, the inclusion body near the pole ends up being captured by the minicell and removed from the mother cell over a period of 40 min. (B) Fate of 43 inclusion bodies near poles that were tracked until the formation of the next minicell. A frequency of 41/43 inclusion bodies was captured by the next minicell. The remaining two were captured by a second minicell at a later time period. *P* < 0.001 by chi-square against a null model of 50% each. *, **, and *** denote *P* values less than 0.05, 0.01, and 0.001, respectively.

To examine whether minicells could preferentially contain aggregates of damaged proteins, we used the heat shock protein *IbpA-yfp* fusion (51) to label and track the aggregates during minicell production. IbpA is a small chaperone that facilitates the refolding of damaged proteins and is therefore associated with inclusion bodies in E. coli (70). We followed the fluorescence of growing single cells at 6 µg streptomycin ml^−1^ (Fig. 5A) and found that inclusion bodies located near a pole were almost always harbored by a minicell that was created at that distal end. Of the 43 polar inclusion bodies that we tracked in Δ*minC IbpA-yfp* bacteria, 41 were captured by a minicell and ejected from the mother cell (Fig. 5B). The two inclusion bodies that were not were captured and ejected the next time that the cell made a minicell. Because other studies have shown that nonpolar inclusion bodies tend to relocate to a pole (51), the eventual fate of most inclusion bodies is therefore ejection via minicell release in the Δ*minC* cells.

### Effect of minicell production on doubling times.

**FIG 6:**
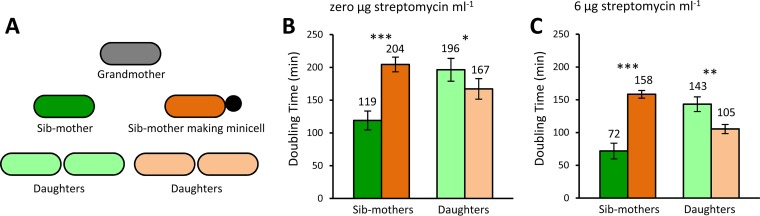
Benefit to daughters of mothers making minicells. Doubling times were obtained by tracking single cells from birth to division into two proliferating cells from the phase-contrast images. The production of minicells was not regarded as division since minicells lack chromosomal DNA and cannot grow and divide. (A) Diagram illustrating the relationship in a lineage of minicell-producing and nonproducing sibling mothers. The sib-mothers have the same mother, which is the grandmother of the lineage (gray). One sib-mother (dark orange) produces a minicell, while the other (dark green) does not. Because the production of a minicell lengthens the doubling time, it should provide no immediate benefit to the mother. To determine if a benefit arises in the next generation, the doubling times of the daughters of minicell-producing and nonproducing sib-mothers were compared (light orange versus light green). (B) Doubling times of sibling mothers and their daughters measured by time-lapse microscopy in growing colonies at 0 µg streptomycin ml^−1^. Minicell-producing sib-mothers (dark orange) had significantly longer doubling times than nonproducing sib-mothers (dark green) (*t* test; *n* = 96; *P* < 0.001). On the other hand, daughters (light orange) of producing sib-mothers had significantly shorter doubling times than daughters (light green) of nonproducing sib-mothers (*t* test; *n* = 192; *P* = 0.043). (C) Same as panel B but at 6 µg streptomycin ml^−1^. Minicell-producing sib-mothers (dark orange) had significantly longer doubling times than nonproducing sib-mothers (dark green) (*t* test; *n* = 90; *P* < 0.001). On the other hand, daughters (light orange) of producing sib-mothers had significantly shorter doubling times than daughters (light green) of nonproducing sib-mothers (*t* test; *n* = 180; *P* = 0.0012). Error bars show standard errors of the means. *, **, and *** denote *P* values less than 0.05, 0.01, and 0.001, respectively. Although daughters of producing and nonproducing sib-mothers had different doubling times, they produced their own minicells at equal rates (see Results for details).

Although the above-described results established a mechanistic link between damage and minicells, we sought next to determine whether the association provided a real benefit. By following growing cells by microscopy at 0 and 6 µg streptomycin ml^−1^, we tracked and recorded the doubling times of sibling mothers (sib-mothers) and their daughters. The release of minicells was not counted as division. Sibling mothers were pairs that had been produced by the same grandmother. We then focused only on sibling pairs in which one sib-mother produced a minicell and the other did not, as exemplified in Fig. 6A. The results revealed that sib-mothers producing minicells had much longer doubling times than the nonproducing sib-mothers at both levels of streptomycin (Fig. 6B and C). These longer times were expected because the producing sib-mothers needed time to make minicells. However, the doubling times of their daughters were totally reversed (Fig. 6B and C). At 0 µg streptomycin ml^−1^, the doubling times of daughters descending from sib-mothers that had produced minicells were 15% shorter (mean of 167 versus 196 min; *P* = 0.044). At 6 µg streptomycin ml^−1^, the effect was even stronger and the doubling times of the same daughters were 27% shorter (mean of 105 versus 143 min; *P* = 0.0012). The weaker but significant 15% effect at 0 µg streptomycin ml^−1^ suggests that aerobic growth in M9 minimal medium generates sufficient damage to provide an advantage to the daughters. A possible concern in interpreting the results is that the longer doubling times of daughters caused by sib-mothers not making minicells may have resulted from a higher rate of minicell production. Because their mothers had not produced minicells, those daughters might be more prone to producing them. To control for that possibility, we compared the numbers of minicells produced by the various daughters. At 0 streptomycin ml^−1^, daughters from nonproducing and producing sib-mothers made 18 and 17 minicells, respectively, and the difference was not significantly different from the null expectation that the two daughters made equal numbers of minicells (*P* = 0.87; chi-square test). At 6 µg streptomycin ml^−1^, the respective number was 48 and 47 minicells, which were also not significantly different from the null expectation (*P* = 0.92; chi-square test). Thus, minicell production cannot account for the doubling time advantage of daughters produced by minicell-producing mothers. Although making minicells hurts the mothers, it benefits the daughters. These results are noteworthy because, besides demonstrating that producing minicells is advantageous, they show that an unknown secondary mutation in the Δ*minC* strain cannot be spuriously providing the advantage. It is the production of minicells that generates the advantage.

We note that the doubling times of the sib-mothers in Fig. 6B were longer than the values reported in Fig. 1A for Δ*minC* cells at 0 µg streptomycin ml^−1^. The reason for the difference is that Fig. 1A encompasses the entire population and the sib-mothers are not a representative sample. Because one member of a pair of sib-mothers produces a minicell, they are a biased subsample. Some cells in the population are not producing minicells, and they will have a shorter doubling time. Thus, the doubling times of the sib-mothers are biased to be longer. The doubling times of the sib-mothers at 6 µg streptomycin ml^−1^ (Fig. 6C) are less biased to be longer because more cells in the population are making minicells at that antibiotic level (Fig. 1C).

### Population benefits for Δ*minC* versus wild-type E. coli.

To determine whether the production of minicells provided any fitness benefits at the population level, we examined next the survival and growth of Δ*minC* cells in cultures over a wider range of streptomycin levels. For a comparison, we used as controls two wild-type strains that did not produce minicells. The two control strains were marked by a *malT* and a *lacA* deletion, respectively, but were otherwise coisogenic members, along with our Δ*minC* strain, of the Keio collection of knockouts. The *malT* and *lacA* deletions were chosen as controls because both are known to have fitness effects that are much smaller than the values that we anticipated for the Δ*minC* strain in glucose minimal medium with streptomycin. The Δ*lacA* deletion has a fitness effect only under very specialized conditions (71), and the Δ*malT* deletion is known to provide only a 1% fitness benefit in glucose minimal medium (72). Our use of two controls offers redundancy for a better estimate of the wild-type response.

**FIG 7:**
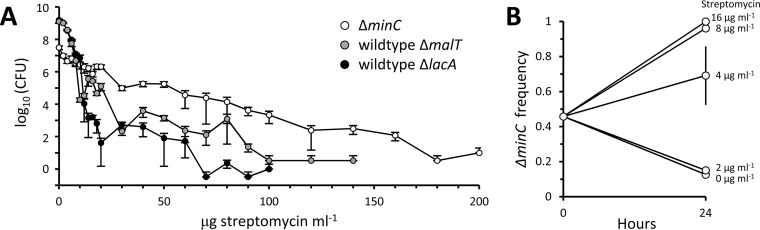
Survival and competition of Δ*minC* and wild-type populations in streptomycin cultures. (A) Cell densities (CFU) of Δ*minC* (open circles) and wild-type Δ*malT* (black circles) strains and Δ*lacA* (gray circles) strain after 24 h of incubation in 1-ml M9 minimal medium monocultures at increasing levels of streptomycin. All monocultures were done in triplicate, and the plotted CFU values are averages. (B) Frequency of Δ*minC* cells after 24 h of incubation in 10-ml M9 minimal medium mixed cultures at increasing levels of streptomycin. The mixed cultures were initiated with a 1:1 ratio of Δ*minC* and wild-type Δ*malT* strains and sampled for CFU before and after incubation. Tetrazolium indicator plates were used to distinguish between Δ*minC* and wild-type Δ*malT* strains, which form white and red colonies, respectively. All mixed cultures were replicated five times, and the plotted frequencies of Δ*minC* cells are averages. The standard error of the mean is presented on the graph for all the averages, but the error bar is visible only for the 4-µg streptomycin ml^−1^ mixed culture. The reason for the larger error at 4 µg ml^−1^ was because that concentration was the threshold at which sometimes the Δ*minC* strain outcompeted the wild-type Δ*malT* strain (increased to nearly 100%) and other times lost.

We first determined the growth and survivorship of monocultures of Δ*minC*, Δ*malT*, and Δ*lacA* strains in the presence of streptomycin concentrations ranging from 0 to 200 µg ml^−1^. Concentrations of 100 to 200 µg ml^−1^ are the working levels generally used when lethal or bactericidal effects are needed to treat wild-type E. coli (73, 74). The growth rate MIC of streptomycin for wild-type strains is approximately 18.5 µg ml^−1^ (75). At concentrations near the MIC, streptomycin increases protein damage and the formation of inclusion bodies (62, 63). After an inoculation of approximately 10^7^ CFU ml^−1^ from overnight cultures free of streptomycin, the monocultures were grown for 24 h and sampled again for CFU ml^−1^. Because minicells do not form colonies, CFU, as opposed to, for example, optical density, provides the most stringent measurement of fitness. Our results showed that while the wild-type controls had an edge at streptomycin concentrations less than a threshold of 10 µg ml^−1^, the Δ*minC* bacteria gained an overwhelming advantage at the higher levels (Fig. 7A). The two wild-type controls responded similarly, suggesting that the Δ*malT* and Δ*lacA* deletions were not affecting the general outcome, at least in comparison to the *minC* deletion. At streptomycin levels below the threshold, the wild-type monocultures were able to increase to overnight densities above the inoculum density of 10^7^ CFU. The threshold value of 10 µg ml^−1^ is close to the wild-type growth rate MIC of 18.5 µg ml^−1^ (75). On the other hand, the Δ*minC* strain was able to proliferate above the inoculum density only at 0 µg streptomycin ml^−1^. The low densities of the Δ*minC* strain are partly explained by the fact that the bacterium is producing minicells, which exact a cost by contributing neither to reproduction nor to CFU. However, despite not being able to proliferate, the Δ*minC* strain was able to persist much better than wild-type bacteria over a wide range of streptomycin concentrations above the threshold.

### Benefits for Δ*minC* strain in mixed cultures.

The above-described monocultures demonstrated a difference in growth and survival of Δ*minC* bacteria from wild-type controls. While the results showed strong differences, a final test of the benefits and costs of minicell production is to compete the two bacteria, side by side, in mixed cultures. The mixed cultures were initiated by inoculating Δ*minC* and Δ*malT* cells from streptomycin-free overnight monocultures, each at an inoculum of 10^7^ CFU ml^−1^, and at a 1:1 ratio. The mixed cultures were then grown for 24 h in 0, 2, 4, 8, and 16 µg streptomycin ml^−1^. The range of streptomycin concentrations was chosen to straddle the 10-µg ml^−1^ threshold observed in Fig. 7A. The 24-h populations were then plated and counted for CFU. Colonies were scored on tetrazolium-maltose indicator plates, which differentiate the competitors by their ability to utilize maltose. While the Δ*malT* strain cannot utilize maltose, this phenotype is under minimal selection in the glucose minimal medium used for the mixed cultures (70) (see “Bacterial strains” in Materials and Methods).

The mixed cultures revealed the same qualitative patterns as the monocultures (Fig. 7B). At the low concentrations of 0 and 2 µg streptomycin ml^−1^, the frequency of Δ*minC* bacteria in the overnight populations decreased from the initial 1:1 ratio in the inocula. At concentrations greater than 4 µg streptomycin ml^−1^, the frequency increased to nearly 100% at the highest values. The concentration of 4 µg ml^−1^ is the transition threshold, and the standard error was the largest because some frequencies increased while others decreased. The standard errors for streptomycin concentrations greater or less than 4 µg ml^−1^ are not shown in Fig. 7B because they were smaller than the point markers. Thus, the qualitative pattern is again that Δ*minC* bacteria are at a disadvantage at the lower concentrations of streptomycin but have an advantage at the higher levels. The transition point of 4 µg ml^−1^ is lower than the 10-µg ml^−1^ threshold observed in the monocultures (Fig. 7A). This difference between monocultures and mixed cultures is not unexpected, as competition in mixed populations cannot be identical to growth and survival in a single population.

**FIG 8:**
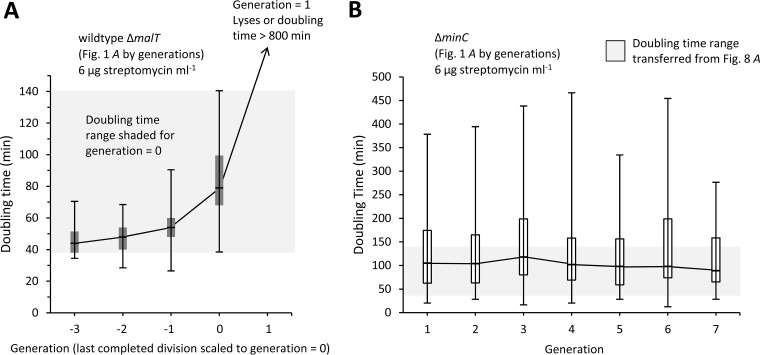
Doubling time of Δ*minC* and wild-type Δ*malT* lineages with streptomycin. Values were extracted from the Fig. 1A (6-µg streptomycin ml^−1^) cells, which were previously pooled but are now sorted by generations. Box plots represent maximum doubling time, third quartile, median, first quartile, and minimum value. Lines connecting the box plots go through the median. (A) Box plots of Δ*malT* doubling times. Generation times for Δ*malT* lineages were rescaled because all of the lineages stopped dividing at this streptomycin concentration, and different lineages stopped at different times. Thus, generation = 0 was set as the point when the last division occurred. As a consequence, all cells at generation = 1 either lysed or failed to divide within an 800-min observation window (limited by the length of the videos). The shaded area highlights the maximum to minimum range of the doubling times observed at generation = 0. Sample sizes from generations −3 to 1 were 6, 17, 45, 94, and 94, respectively. The slope of a linear regression of the doubling times onto generations was significantly greater than zero (*P*  < 0.001; *r*^2^ = 0.34; excluding generation = 1). (B) Box plots of Δ*minC* doubling times. The shaded area covers the same doubling time range as in panel A and corresponds to the maximum to minimum range of doubling times of the Δ*malT* strain box plot at generation = 0. Sample sizes from generations 1 to 7 were 12, 35, 43, 51, 50, 43, and 38, respectively. Generations 8, 9, and 10 were not included or presented because their sample sizes were too small.

The different survival and persistence of the Δ*minC* and wild-type Δ*malT* cells in the monocultures and mixed cultures can be quantified at the single-cell level by separating the cells from Fig. 1A (6 μg streptomycin ml^−1^) and sorting them by generation. However, the generation times of the Δ*malT* lineages had to be rescaled because they all stopped dividing after a few generations and at different time points. Thus, all Δ*malT* lineages were rescaled to have generation = 0 as the last time point before division stopped at 6 μg streptomycin ml^−1^ (Fig. 8A). These Δ*malT* lineages doubled with times that increased from 47 to 82 min from generation = −3 to 0, and all stopped dividing by generation = 1. The slope of the increase in doubling times was significantly greater than zero by a linear regression (*P* < 0.001; *r*^2^ = 0.34; excluding generation = 1). Cells that stopped dividing at generation = 1 either lysed or had division times that exceed our 800-min inspection window, which was limited by the duration of our videos. On the other hand, the Δ*minC* lineages were able to sustain steady growth and cell division for many more generations up to the end of the videos. However, Δ*minC* doubling times are presented for only the first seven generations (Fig. 8B) because video lengths varied and the sample sizes for higher generations were small. The slope of a linear regression of Δ*minC* doubling times onto generations was not significantly different from zero (*P* = 0.32; *r*^2^ < 0.0001), which indicates that the doubling times were not changing over generations. It is noteworthy that at generation = 0 the box plot range (maximum to minimum) for the Δ*malT* doubling times, which is highlighted by shading in Fig. 8A and B, increased to be totally included in the box plot range of the Δ*minC* lineages. Thus, as their doubling times lengthened, many wild-type Δ*malT* cells acquired the same doubling times as Δ*minC* cells at 6 μg streptomycin ml^−1^.

## DISCUSSION

Our study shows that Δ*minC* E. coli not only produced minicells at a higher rate than wild-type strains but also increased the production rate in the presence of streptomycin (Fig. 1C). Additionally, we found that the production of minicells was more often associated with the old poles and old daughters of the bacteria (Fig. 3G and H). Because aggregates of damaged proteins or inclusion bodies in E. coli relocate to the old pole, they were invariably contained by minicells and ejected from the mother bacterium (Fig. 5A and B). Minicells were also found to take the old cell wall from the old pole rather than synthesizing a new structure (Fig. 4). The production of minicells incurred a cost by lengthening the doubling time of the mother (Fig. 1A). However, minicell production and the *minC* deletion were also found to provide advantages. In Δ*minC* E. coli, the daughters of mothers that produced minicells had shorter doubling times than daughters of nonproducing mothers (Fig. 6B and C). This can be seen as rejuvenation, the opposite of aging. Because mothers produce two daughters, the benefit of producing daughters that grow faster is amplified. Additionally, compared to wild-type strains, Δ*minC* E. coli persisted or survived better at intermediate and higher levels of streptomycin (Fig. 7A). At those concentrations of streptomycin, the benefits were found to outweigh the costs because Δ*minC* E. coli had a competitive advantage over the wild-type strain in mixed populations (Fig. 7B). Although streptomycin is bactericidal, cells can survive and recover from exposures to the antibiotic (Fig. 7A), and Δ*minC* E. coli clearly has a higher tolerance than wild-type bacteria.

These combined results show that minicells provide an advantage to bacteria as a disposal mechanism for damaged proteins. Given the recent growing recognition that damage has a strong deleterious effect on bacterial replication and aging (51–54, 64–67, 76), such a role for minicells becomes important. The advantage that minicells provide in the presence of streptomycin suggests that they could also play a role in helping bacterial cells resist, survive, or persist when challenged with antibiotics (Fig. 7A). It remains to be determined whether the survival of the Δ*minC* cells at the higher streptomycin levels is due to dormancy, as in the stricter definition of persisters that survive drug treatment (77, 78). By eliminating damage, minicells could help bacteria survive by sustaining growth at a low level and without going into a state of dormancy. However, minicells could also help bacteria become better persisters, such as entering or exiting dormancy more safely. Our doubling time data at 6 µg streptomycin ml^−1^ (Fig. 8A and B) suggest that the higher sensitivity of the wild-type cells, relative to Δ*minC* cells, cannot be explained by the longer doubling times of the Δ*minC* cells. In the same manner that dormancy protects persisters, it could be that longer doubling times are protective. However, our results showed the opposite. The doubling times of wild-type Δ*malT* lineages at 6 µg streptomycin ml^−1^ increased over generations, but the increase, rather than being protective, led to the death of the lineage (Fig. 8A). Additionally, the doubling times of these wild-type cells had lengthened to match many of the Δ*minC* doubling times at the same concentration of streptomycin. If the minimum and maximum doubling times of the Δ*minC* cells are identified from Fig. 8B box plots, they contain 100% of the wild-type Δ*malT* doubling times from the last generation before the lineage stopped dividing (Fig. 8A, generation = 0; shaded area). However, despite their overlapping doubling times, all of the Δ*minC* cells were able to sustain growth and produce viable daughters, while all of the Δ*malT* cells at that last generation produced nondividing daughters. The generality of the effect of minicells in response to streptomycin needs also to be examined for other antibiotics. If the response is general, our results could be uncovering a new mechanism that is used by bacteria to resist antibiotics.

The use of Δ*minC* E. coli to study the benefits of producing minicells, when laboratory wild-type bacteria produce them at a very low rate, raises the question of whether using a hypermutation is an appropriate approach. Because the cost of making minicells is high (Fig. 1A), it is expected that any standard laboratory wild-type strain would have been selected not to make minicells. Thus, the only option available to us was to use a hypermutation such as Δ*minC*. Augmenting a phenotype to assess its consequences is not necessarily a less conservative approach. If hyperexpressing minicells were overly deleterious, because of unknown pleiotropic side effects, the outcome of our experiments would have been different. The use of a hypermutation to assess the benefit of a phenotype has precedence. The original studies that investigated the benefits of mutation rates in E. coli employed mutator genes with 100-fold-higher rates than wild-type laboratory strains (55–57). They showed that mutator alleles provided an advantage when bacteria were confronted with novel environments. However, the elevated rate incurred a cost (57), which explains why wild-type strains grown under more constant laboratory conditions have low mutation rates. However, if high mutation rates and minicell production are beneficial but costly, why has wild-type E. coli evolved not to express those phenotypes as a regulated and inducible response? Wild E. coli from natural populations may be inducible, but it is also possible that the *minC* locus has evolved more like mutator loci. Long-term evolution in E. coli populations has revealed that mutation rates can evolve genetically from low to high and back to low over generations (58). Supporting this premise is the fact that many wild E. coli strains have mutator alleles and others show genetic patterns that suggest a back-and-forth evolution from wild-type to mutator alleles (59). Thus, although elevated mutation rates can be beneficial, E. coli has evolved not to change the rate as a regulated and inducible response. If mutator alleles serve as an example, perhaps minicell production is another phenotype in E. coli that evolves back and forth through mutations, rather than by an inducible response.

A revived interest in bacterial minicells has come from the recent growth in their use as nano-sized delivery system for drugs, vaccines, or other payloads to targeted cells or tissues (21–32). Besides the major benefit of having few or no side effects, the other advantages of minicells are their small and uniform size and ease of production, delivery, and uptake. In cancer treatments, minicells are collected, loaded with the appropriate drug, tagged with antibodies for a target cancer, injected into the blood system, attached to the target cells through the antibodies, and taken up into the cells by endocytosis. The loading of the payload is currently all done by diffusion during coincubation with minicells. Our demonstration that inclusion bodies are invariably contained by minicells suggests that there is an intracellular route to loading minicells. If a payload could be synthesized by Δ*minC* bacteria, it could be loaded intracellularly. An intracellular route is attractive for payloads that, perhaps because of permeability problems, cannot be loaded extracellularly by diffusion and coincubation. Intracellular loading could be done passively by having the payload near the old poles of cells. Alternatively, if there are features or signals that relocate inclusion bodies to the old poles or minicells, they could be used to direct the payload. For example, if misfolded or damaged peptides are also relocated to inclusion bodies, they could be linked to the payload.

## MATERIALS AND METHODS

### Bacterial strains.

Three E. coli strains, Δ*minC*, Δ*malT*, and Δ*lacA* knockouts, from the Keio collection (60) were used in this study. The Keio collection was created for the analysis of single-gene functions and mutation effects in an E. coli strain (K-12) and consists of precisely defined, single-gene deletions of all nonessential genes. Because all of the Keio knockouts were created by the same λ Red recombinase protocol and derived from the same parental E. coli BW25113 strain, they are otherwise isogenic and all contain Kan^r^ in place of the knocked-out gene. The isogenicity of the knockout mutants makes the Keio strains ideal for comparing the effects of knockouts and serving as complementary controls for the loss of function. The most common secondary mutations caused by the λ Red recombinase system are partial duplications, but all of the Keio strains have been checked, and 98.3% of them, including Δ*minC*, Δ*malT*, and Δ*lacA* knockouts, have been verified not to harbor any (61). The Δ*malT* and Δ*lacA* strains were chosen to serve as wild-type controls because they had a functional *minC* allele and their respective deletions are known to have minimal fitness effects (71, 72). The Δ*malT* allele was used to differentiate the strains when cocultured with the Δ*minC* strain. The Δ*malT* and Δ*lacA* strains were also better controls than the parental BW25113 strain because the latter lacked the Kan^r^ insert found in all Keio knockouts. The absence of the Kan^r^ insert, which is likely burdensome, could have given BW25113 a false advantage. The fluorescent protein fusion *IbpA-yfp-*Cm^r^ used to visualize inclusion bodies was obtained from a construct by Ariel Lindner (INSERM, France) (51). The construct was amplified by PCR and inserted into the chromosome of Δ*minC* via the λ Red recombinase system (79).

### Culture and agar plate media.

All cultures were grown with M9 glucose minimal medium (80) with the addition of 0.2 mg thiamine ml^−1^ (Sigma). When required, streptomycin (Hoechst) was added at the indicated concentrations. CFU were obtained by plating on LB and/or TTC (triphenyl tetrazolium chloride; MP Biomedicals LLC) plates (57, 81). TTC plates supplemented with maltose (Sigma-Aldrich) were indicator plates that were used to distinguish between Δ*minC* and Δ*malT* strains, which form white and red colonies, respectively, based on their ability to ferment maltose. Plates and cultures were incubated overnight at 37°C. All cultures were aerated with shaking.

### Growing and visualizing cells by microscopy.

To track the *in vivo* growth of dividing single cells and lineages, bacteria were tracked by time-lapse microscopy. Cells were grown on agar pads (64, 67) containing 15 mg ml^−1^ of electrophoresis-grade agarose (Fisher Scientific) in M9 medium with the desired concentration of streptomycin. All cells used to inoculate agar pads were grown exponentially in streptomycin-free M9 medium. Time-lapse images were capture on a Nikon Eclipse Ti microscope with the Nikon NIS Element AR software by 100× phase-contrast and fluorescence (Prior, Lumen 200) imaging. To visualize the distribution of cell wall during the formation of minicells, cells were pulse-labeled with Alexa Fluor 488 wheat germ agglutinin (WGA) (Life Technologies Corporation) (68). Phase-contrast images were taken at intervals of 2 min. To minimize phototoxicity and bleaching, fluorescence images were recorded at longer intervals of 10 and 30 min. Microscope and agar pads were kept at 37°C with a Nevtek ASI 400 heater.

### Data collection from time-lapse images.

All images were analyzed with ImageJ (NIH). Doubling times were obtained by tracking single cells from birth to division into two proliferating cells from the phase-contrast images. The production of minicells was not regarded as division since a minicell does not contain chromosomal DNA and cannot grow and divide. The release of a minicell is easily distinguished from a cell division by the fact that the minicell is consistently released from the bacterial pole as a sphere with a diameter about equal to the width of a rod-shaped E. coli cell. A minicell also does not grow or change in shape and is very stable over our observation periods of up to 24 h. Old and new daughters and poles were determined by tracking lineages over two generations as indicated in Fig. 2. Note that the assignment of the first cell in Fig. 2 can be unknown. However, its assignment can be determined if three or more generations can be linked. The counting of frequency of minicells to whole cells (Fig. 1C) was restricted to colonies smaller than 50 cells to avoid the crowding and masking of minicells.

### Survival and competition experiments.

The survival of the Δ*minC*, Δ*malT*, and *ΔlacA* E. coli strains in monocultures was determined in 1-ml cultures that were initiated with approximately 10^7^ CFU ml^−1^ from streptomycin-free overnight cultures. The 1-ml cultures contained M9 minimal medium with streptomycin levels ranging from 0 to 200 µg ml^−1^ (Fig. 7A). After 24 h of incubation, the monocultures were sampled for CFU counts on LB plates. All monocultures were replicated three times and averaged at each streptomycin concentration. Competition experiments were conducted in mixed cultures with only Δ*minC* and Δ*malT* strains in 10 ml of M9 at concentrations of 0, 2, 4, 8, and 16 µg ml^−1^ (Fig. 7B). The mixed cultures were initiated with approximately 10^7^ CFU ml^−1^ of each competitor from streptomycin-free overnight cultures. After 24 h of incubation, the mixed cultures were sampled for Δ*minC* and Δ*malT* CFU counts by plating on TTC-maltose indicator plates. All mixed cultures were replicated five times. Growth and survival were always measured by CFU counts, rather than by optical density, because minicells, although inviable, still contribute to the density and would lead to an overestimate of the replicative potential of the bacterial population.

### Statistical analysis.

Sample sizes are provided in either the figures or the figure legends. Tests of significance between two data sets were conducted by paired and unpaired *t* tests as required by the structure of the data. For example, a paired test was used when comparing siblings or daughters that constituted a pair matched to the same mother (Fig. 6B and C). Chi-square tests were used to determine if the production of minicells by old versus new poles or daughters (Fig. 3G and H) deviated from a null model of equal contribution. For comparing the abundance of minicells to whole cells in colonies (Fig. 1C), a chi-square test could not be used because the sample sizes were unequal. Thus, the minicell-versus-whole-cell difference at the 0- and 6-µg streptomycin ml^−1^ treatments was determined by a randomized test of relative frequencies by resampling with the statistical package StatKey (http://www.lock5stat.com/StatKey/). All estimated *P* values are presented in the figure legends and as qualitative thresholds in the figures (*, **, and *** denoting *P* values of less than 0.05, 0.01, and 0.001, respectively).
